# Higher Serum Monolaurin Is Associated with a Lower Risk of COVID-19: Results from a Prospective Observational Cohort Study

**DOI:** 10.3390/ijms26062452

**Published:** 2025-03-10

**Authors:** Daniele Sola, Stelvio Tonello, Giuseppe Francesco Casciaro, Eleonora Rizzi, Davide D’Onghia, Mario Pirisi, Francesca Caldera, Manuela Rizzi, Donato Colangelo, Nicoletta Del Duca, Massimo Scacchi, Elia Amede, Denise Marradi, Elettra Barberis, Annalisa Chiocchetti, Marcello Manfredi, Pier Paolo Sainaghi

**Affiliations:** 1 IRCCS Istituto Auxologico Italiano, Laboratory of Metabolic Research, S. Giuseppe Hospital, Piancavallo, 28824 Oggebbio, Italy; d.sola@auxologico.it (D.S.); n.delduca@auxologico.it (N.D.D.); massimo.scacchi@unimi.it (M.S.); 2 Department of Translational Medicine, Università del Piemonte Orientale, 28100 Novara, Italy; stelvio.tonello@med.uniupo.it (S.T.); davide.donghia@uniupo.it (D.D.); mario.pirisi@uniupo.it (M.P.); elia.amede@uniupo.it (E.A.); denise.marradi@uniupo.it (D.M.); marcello.manfredi@uniupo.it (M.M.); 3 Department of Internal Medicine and COVID-19 Unit, AOU “Maggiore della Carità”, Via Mazzini 18, 28100 Novara, Italy; fra.casciaro90@gmail.com (G.F.C.); eleonora.rizzi18@gmail.com (E.R.); francesca.caldera@maggioreosp.novara.it (F.C.); 4 Cancer Research Institute, Beth Israel Deaconess Medical Center, Harvard Medical School, Boston, MA 02215, USA; 5 Department of Health Sciences, Università del Piemonte Orientale, Via Solaroli 17, 28100 Novara, Italy; manuela.rizzi@uniupo.it (M.R.); annalisa.chiocchetti@uniupo.it (A.C.); 6 Department of Health Sciences (Department of Excellence 2023–2027), Pharmacology, Università del Piemonte Orientale, Via Solaroli 17, 28100 Novara, Italy; 7 Department of Clinical Sciences and Community Health, Università di Milano, 20122 Milan, Italy; 8 Department of Science and Technological Innovation, Università del Piemonte Orientale, 15121 Alessandria, Italy; elettra.barberis@uniupo.it

**Keywords:** COVID-19, monolaurin, metabolomic

## Abstract

The COVID-19 pandemic has stimulated the search for effective preventive and therapeutic agents. In recent years, many studies have considered the effects of different nutrients. This study aimed to investigate the association between serum monolaurin levels and the risk of developing COVID-19 among healthcare workers. In this prospective observational cohort study, 2712 healthcare workers from the University Hospital “Maggiore della Carità” in Novara, Italy were enrolled. Participants underwent blood sampling and were followed up for six months to evaluate the protective role of serum monolaurin against COVID-19 infection. Monolaurin levels were quantified using targeted metabolomic analysis. The study cohort consisted of 1000 individuals with a mean age of 46.4 years, predominantly female. Higher serum monolaurin concentrations were significantly associated with a lower risk of SARS-CoV-2 infection at both 3- and 6-month follow-ups. The optimal cut-off value for serum monolaurin, which provides protective efficacy, was identified as 0.45 µg/mL. Higher serum monolaurin levels appear to be associated with a reduced risk of COVID-19, suggesting its potential as a protective dietary supplement against SARS-CoV-2 infection. This study contributes to the growing body of evidence supporting the role of dietary factors in the management and prevention of infectious diseases and highlights the potential of targeted metabolomics in identifying prophylactic biomarkers.

## 1. Introduction

At the end of 2019 a cluster of pneumonia cases of unknown origin was first reported in the province of Wuhan (China), and Chinese scientists identified the severe acute respiratory syndrome coronavirus-2 (SARS-CoV-2) as the etiological agent of the novel coronavirus disease 2019 (COVID-19). SARS-CoV-2 is a positive-sense, single-stranded, RNA virus showing a high genetic similarity with both SARS-CoV and MERS-CoV, the viruses responsible for the previous coronavirus pandemic outbreaks [1,2]. SARS-CoV-2 is an enveloped virus, characterized by the presence of structural spike proteins, directly involved in viral infection, through their interaction with receptors (i.e., angiotensin converting enzyme 2—ACE2) and coreceptors (i.e., heparan sulphate proteoglycans) exposed on the host cell membrane [1,2,3].

The clinical spectrum of COVID-19 manifestations is wide, ranging from asymptomatic/paucisymptomatic cases to severe/critical cases requiring hospital admission and intensive care support. Furthermore, due to the involvement of different organ systems, in critically ill patients, the risk of death is still not negligeable even if severe cases are now very rare [3,4].

While at the beginning of the pandemic, the clinical treatment of infected patients was almost supportive and non-specific (i.e., corticosteroids and anticoagulants), to date, the number of therapeutic options available to fight the infection has increased. The most commonly used anti-COVID-19 options are mainly represented by antiviral agents (i.e., remdesivir, molnupiravir, and paxlovid) and biological immunomodulators (i.e., tocilizumab, anakinra, anti-SARS-CoV-2 neutralizing antibodies), whose efficacy is highly dependent on the disease stage and on the circulating viral variant at the time of infection and treatment [2,5]. In spite of the mass vaccination campaign role in reducing severe COVID-19, the in-hospital mortality for unvaccinated subjects remains quite high [6,7], thus fostering the search for new targeted therapeutic approaches as well as for preventive agents able to reduce infection risk.

In this scenario, nutritional supplements grew in popularity, due to their large availability, safety, and presumed antiviral efficacy or immunomodulatory effects [8,9,10,11,12]. Moreover, as everyone’s diet is a modifiable factor influencing the risk of developing several infectious diseases, many dietary recommendations have been issued in relation to the COVID-19 pandemic, with a special attention on those nutrients supposed to boost the immune system and to reduce inflammation (i.e., vitamin C, vitamin D, zinc, selenium, and fatty acids). Several papers have shown that a balanced intake of macro- and micro-nutrients correlates with disease prevention and better outcomes sustaining immune defenses against both SARS-CoV-2 and opportunistic infections [13,14].

Among these nutritional supplements that are supposed to exert a beneficial role in preventing and/or reducing SARS-CoV-2 infection or in mitigating COVID-19 manifestations, coconut oil is an option. It is edible oil that is largely used in cooking and bakery, and in infant foods. Its proposed role as a health supplement is mainly related to its content in medium-chain fatty acids, which have been proved to exert anti-obesity, anti-inflammatory, and antimicrobial/antiviral activities [15,16]. Among the medium-chain fatty acids present in coconut oil, the most studied, due to their recognized activity as both antimicrobial/antiviral and immunomodulating agents, are lauric acid and its derivative monolaurin [15,17,18,19,20]. Monolaurin (also known as glycerol monolaurate) is the monoester of glycerol and lauric acid and is particularly abundant in coconut oil as well as in breast milk [17,18,19,20,21,22]. Monolaurin is a generally recognized as safe (GRAS) compound according to the US Food and Drug Administration (FDA). It shows an effective antimicrobial effect against a wide range of Gram-positive and Gram-negative microorganisms, bacterial biofilms as well as against different enveloped viruses, thus supporting its clinical use through topical application (i.e., mouthwashes and vaginal tampons) [17,19,20,21,22,23].

Some published papers reported a potential antiviral effect of monolaurin also against SARS-CoV-2 [19,24], thus fostering further studies dealing with its potential use in the context of the ongoing pandemic. In particular, in a previously published work [25] based on a small cohort of healthcare professionals, it has been demonstrated, through an untargeted metabolomic approach, that circulating monolaurin and cholesterol levels were related to SARS-CoV-2 infection risk. As a matter of fact, in the study, it has been demonstrated that the subjects showing higher levels of circulating monolaurin were less likely to develop SARS-CoV-2 infection in the three weeks following the study blood draw, while those with higher cholesterol levels were at higher risk of developing COVID-19. Even if those data were interesting, the main limitation of this previous study was represented by the small number of subjects involved. To better elucidate the protective role of monolaurin in protecting from SARS-CoV-2 infection, in this study, we performed a targeted metabolomic analysis on a larger cohort of healthcare workers.

## 2. Results

### 2.1. Characteristics of the Population

**Figure 1:**
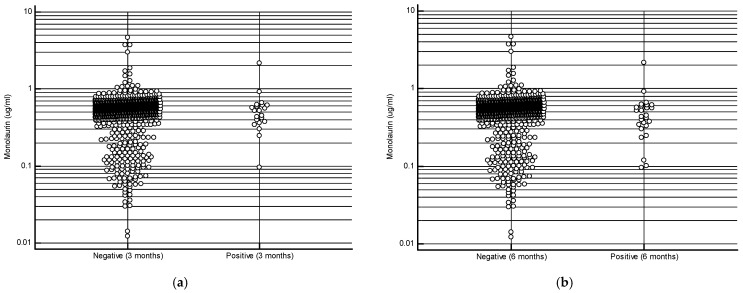
Monolaurin values in patients who developed or did not develop the infection at 3 months (**a**) and at 6 months (**b**).

The study cohort consisted of 1000 subjects with an average age of 46.4 ± 11.5 years, with a female predominance (70.5%). The most frequent comorbidities observed in our cohort were hypertension, thyroid diseases, and a history of neoplasia (Table 1). At the 3-month follow-up, 979 subjects (97.9%) tested negative for SARS-CoV-2 infection, a result similar to that observed at the 6-month follow-up (974 negative subjects—97.4%). The mean serum monolaurin concentration was significantly higher in uninfected compared to infected individuals at both time points: 0.542 ± 0.303 µg/mL vs 0.563 ± 0.29 µg/mL at 3 months (*p* = 0.02); 0.5440 ± 0.297 µg/mL vs 0.5066 ± 0.391 µg/mL at 6 months (*p* = 0.02) [Figure 1a,b]. As shown in Table 2, the most common symptoms observed in infected individuals included rhinorrhea/nasal congestion, altered taste/smell, and pharyngodynia. No infected subjects required hospitalization.

### 2.2. Analysis of the Impact of Monolaurin on SARS-CoV-2 Infection

**Figure 2:**
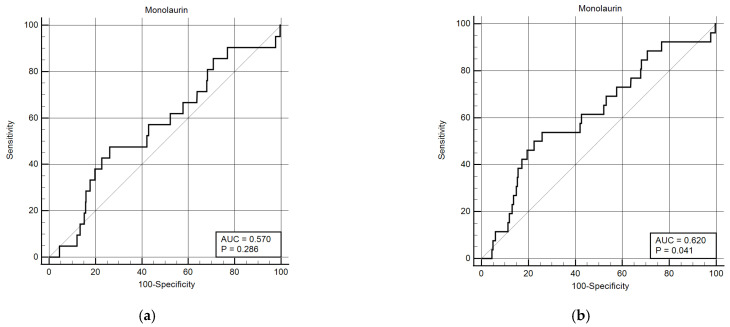
ROC curves for monolaurin values (ug/mL) at 3 (**a**) and 6 months (**b**). The curve was constructed assuming the presence or absence of infection within the 3- and 6-month time points and the monolaurin concentration values. The Youden index for both time points is 0.45 ug/dL.

**Figure 3:**
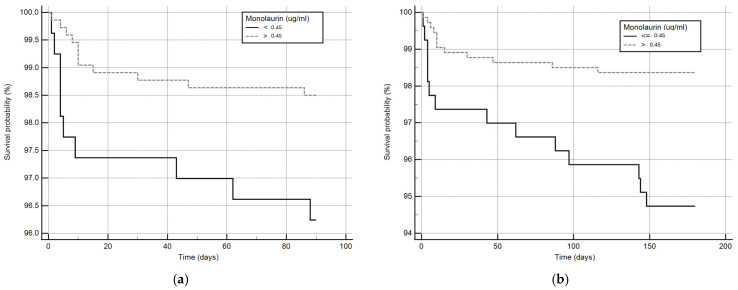
Kaplan–Meier survival curves at 3 (**a**) and 6 months (**b**). The solid line represents patients with circulating monolaurin values lower than 0.45 ug/mL and the dashed line subjects with values higher than the cut-off.

**Figure 4:**
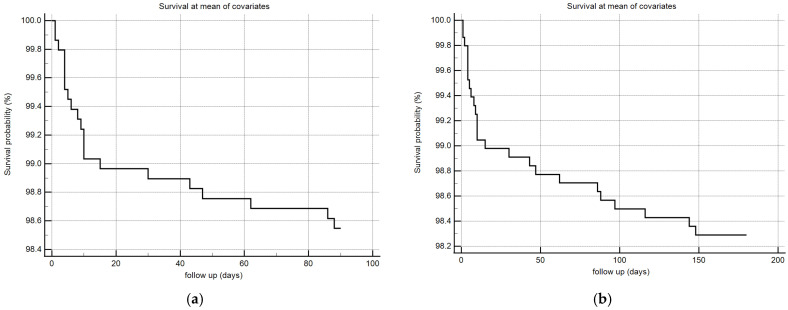
COX survival model plot at 3 (**a**) and 6 (**b**) months, the follow-up is expressed in days (x-axis) and in % survival probability (y-axis).

Considering that monolaurin levels were significantly higher in patients who did not develop SARS-CoV-2 infection at all time points, we built the corresponding ROC curves in order to identify a potential cut-off value. This proved to be crucial for building Kaplan–Meier survival curves, thereby offering new insights for clinical monitoring and therapeutic interventions during the course of the infection. According to ROC curves, the most effective value allowing the discrimination of the infection risk was 0.45 µg/mL (Youden Index), with an AUC of 0.57 (CI: 0.538–0.601) at 3 months and of 0.62 (CI: 0.589–0.650) at 6 months (Figure 2). Using the established cut-off value of 0.45 mcg/mL, odds ratios were calculated to assess the risk of infection. The obtained results indicated a significantly higher risk of infection in individuals with monolaurin concentrations below the 0.45 mcg/mL threshold, with an odds ratio of 2.562 (95% CI: 1.077–6.112) at 3 months and 3.34 (95% CI: 1.525–7.323) at 6 months. Based on these observations, we then built Kaplan–Meier survival curves with a monolaurin cut-off of 0.45 µg/mL. As shown in Figure 3a,b, we observed a significant difference at both 3- and 6-month follow-ups (*p* = 0.02 and *p* = 0.001, respectively). These data suggest that a serum monolaurin concentration greater than 0.45 µg/mL may be associated with a lower risk of SARS-CoV-2 infection in the short term (3–6 months follow-up). Kaplan–Meier survival curves were used to assess the influence of several variables including age (categorized as the arithmetic mean), sex, diabetes, autoimmune diseases, liver or renal disorders, arterial hypertension, dyslipidemia, thyroid abnormalities, celiac disease, multiple sclerosis, cardiovascular diseases, neoplasms, and epilepsy. As shown in Table 3, none of the analyzed variables showed a significant effect. Subsequently, a Cox proportional hazards model was built for further verification, incorporating the monolaurin threshold of 0.45 mcg/mL, sex, age (as a continuous variable), and all potential comorbidities. Notably, monolaurin was identified as the only variable significantly associated with the risk of infection at both 3- and 6-month follow-ups. The full model demonstrated a significant improvement over the null model, with the negative log likelihood decreasing from 344.409 to 336.988 at the 3-month follow-up. This was supported by a chi-square value of 7.421 (df = 1, *p* = 0.0064), indicating a significantly better fit with the inclusion of covariates. Continuing to the 6-month follow-up, the negative log likelihood further decreased from 289.382 to 285.043, accompanied by a chi-square of 4.340 (df = 1, *p* = 0.0372), suggesting that the model continued to fit better than the null model over a longer period. Regarding the specific results for monolaurin, identified by the Youden Index cut-off at 0.45 µg/mL, at 3 months the coefficient for monolaurin was −1.1096, with a standard error of 0.4003 and a Wald statistic of 7.6833 (*p* = 0.0056), indicating a hazard ratio (Exp(b)) of 0.3297. This suggests a substantial 67.03% reduction in the relative risk of infection, with the 95% confidence interval ranging from 0.1504 to 0.7225. At 6 months, the coefficient was slightly less at −0.9327, with a standard error of 0.4369 and a Wald statistic of 4.5569 (*p* = 0.0328). The hazard ratio stood at 0.3935, implying a 60.65% reduction in risk, with the 95% confidence interval between 0.1671 and 0.9265, indicating consistent protective effects of monolaurin over time [Table 4 and Table 5], [Figure 4a,b].

## 3. Discussion

Metabolomics is an exciting emerging approach in translational research on infectious diseases, including SARS-CoV-2 infection. Metabolomic analysis of serum samples has provided valuable information for the identification of potential protective factors, risk factors, and biological markers associated with specific disease conditions. The application of this technology in the field of nutrition may be useful for identifying potential molecules that play significant roles in the prophylaxis or treatment of infections, a field of research that has not yet been fully explored. Furthermore, metabolomics represents a promising approach for future research also outside the context of infectious diseases.

This study stems out from the results of a preliminary study, conducted by applying non-selective metabolomic techniques, showing that high levels of serum monolaurin could be a potential protective factor against SARS-CoV-2 infection [25]. Based on this evidence, the present study aimed to confirm, in a larger cohort, these preliminary observations on the protective effect of high serum monolaurin levels. Our results show a significant difference in terms of serum monolaurin levels between subjects who developed SARS-CoV-2 infection and those who remained negative. Indeed, at both observation time points, monolaurin levels were higher in those who did not develop the infection. Based on initial observations, we constructed ROC curves to identify the most effective cut-off value for serum monolaurin that could indicate a potential protective effect. In our cohort, the serum monolaurin concentration corresponding to the Youden index was found to be 0.45 µg/mL. Concentrations exceeding this threshold demonstrated a protective effect against infection at both time points, as evidenced by Kaplan–Meier survival analysis and Cox regression models. In particular, the variable of sex was also taken into account in our analysis due to a higher prevalence of women in our cohort, resulting from the consecutive and random inclusion of healthcare workers from our university hospital. In our Italian context, there is a predominance of women among nurses, social-health workers, and students in the specialization program in Internal Medicine. Using this cut-off, monolaurin concentration retains its statistical significance in the Cox multiple survival analysis including all comorbidities present in the study cohort, despite the significant difference in the number of subjects free from disease and those who contracted the infection. This study was deliberately conducted with a relatively short follow-up to ensure a certain stability of the monolaurin concentration values, which, being dependent on nutrition, fluctuate over time. We believe that a plasma monolaurin concentration value at time 0 (at the time of recruitment) can remain stable and therefore be reliable in predicting possible SARS-CoV-2 infection for a limited number of months.

The antiviral and immunomodulatory properties of monolaurin are well documented, with several recent studies confirming its natural antibiotic/antiviral effect. In an in vitro model, Ghany and colleagues demonstrated the antibacterial activity of monolaurin against *S. aureus* as well as its synergistic activity when administered with β-lactam drugs [26]. In addition, Shi and colleagues showed a 80% inhibition of Seneca Valley virus replication mediated by monolaurin in vitro and a reduction in viral load and associated organ damage in vivo [17].

Monolaurin is abundantly present in virgin coconut oil and in various dietary supplements. The idea of using monolaurin as a supplement is intriguing; however, to date, there is no in vivo evidence to support its use in humans as a dietary supplement. The US FDA recognizes monolaurin as a safe molecule; however, there are no guidelines regarding its standard dosages. To date, the only information available refers to the topical formulation, for which the FDA considers as safe concentrations up to 100 mg/mL [27]. Searching the scientific literature, we could find only a few studies on the use of monolaurin in humans. These studies have focused on topical uses, such as mouthwash and vaginal application [20]. These studies showed a reduction in toxic shock syndrome toxin 1 (TSST-1), interleukin 8 production, and a decrease in the number of various Candida and Gardnerella vaginalis species following vaginal application of monolaurin [28,29] and a reduction in oral Helicobacter pylori infection associated with improved gastric clearance following monolaurin treatment [30].

Considering this promising evidence and the observation of the lack of any potential serious adverse events in studies focused on virgin coconut oil, it is noteworthy that well-designed intervention studies are necessary to confirm and expand the knowledge about safety and the beneficial role of monolaurin supplementation [31].

To our knowledge, this is the first study to report monolaurin levels in the serum of human subjects, so these values, also due to the size and characteristics of the population studied, could be proposed as reference values in the Italian population. While there are no direct precedents in the literature concerning monolaurin’s impact on COVID-19, significant findings related to coconut oil have been documented. Recent studies have revealed the potential therapeutic benefits of virgin coconut oil in managing COVID-19. Angeles-Agdeppa et al. (2021) found that it significantly reduced C-reactive protein levels in SARS-CoV-2-infected subjects, suggesting a potential role in attenuating COVID-19 inflammation [31]. Other research further supports this observation, demonstrating that virgin coconut oil supplementation not only alleviated symptoms but also reduced inflammation in COVID-19-positive adults, highlighting its potential usefulness in symptom management [32]. Furthermore, Alejandria and colleagues (2024) used virgin coconut oil as an adjunctive therapy in hospitalized COVID-19 patients, achieving clinically significant outcomes, thus reinforcing the beneficial effect of this molecule in COVID-19 treatment [33]. Although our study focuses on the protective effect of monolaurin against COVID-19 infection, it is well known that some biological markers, especially interleukin-6 (IL-6), are strongly associated with adverse outcomes in SARS-CoV-2 infections [34], and there is evidence to suggest that monolaurin exerts both direct and indirect effects in reducing the synthesis of inflammatory cytokines [35], potentially leading to more favorable outcomes during infection. However, further research is needed to fully understand these dynamics.

The proposed study suffers from some limitations. First, only Caucasian subjects were enrolled, compromising the generalizability of the results on a multi-ethnic scale, and there is a prevalence of female gender, although this variable was considered in the statistics and did not affect the results. Secondly, only subjects negative for anti-SARS-CoV-2 antibodies at the time of enrollment and who did not have a previously confirmed infection were included in this study. This choice, although essential to ensure the accuracy of this study, implies a selection bias, thus limiting the generalizability of the results to people with previous infections or different immunity status. Third, the follow-up lasted up to 6 months, which seems an appropriate timeframe to observe the protective effect of monolaurin; nevertheless, changes in social behavior, the introduction of new health policies, and/or the emergence of new viral variants could influence the results on longer follow-ups. Finally, monolaurin was quantified only in serum, and these results may not fully reflect the dynamics of monolaurin–virus interaction in the whole body, especially at the mucosal level, which is the main entry route of the SARS-CoV-2 virus. In conclusion, based on the results obtained, it is conceivable that the presence of higher serum monolaurin levels is associated with a lower risk of SARS-CoV-2 infection. Therefore, in our study cohort, a serum concentration higher than 0.45 µg/mL seems to be a protective factor against SARS-CoV-2 infection in the short term (3–6-month follow-up). To the best of our knowledge, this is the first study specifically investigating the protective role of monolaurin in COVID-19 and could represent an interesting starting point for the development of new studies focused on the protective effects of monolaurin against infectious diseases. Furthermore, the proposed metabolomic approach offers new perspectives in the identification of potentially protective biomarkers in various pathological conditions, paving the way for new advancements in the field of personalized medicine.

## 4. Material and Methods

### 4.1. Study Design and Population

In this prospective, observational cohort study, we enrolled 2712 volunteers working at “Maggiore della Carità” University Hospital in Novara (Novara, Italy). This study was approved by the local Ethical Committee (CE 96/20, HCP-COVID-19 study) and conducted according to the Declaration of Helsinki and the Good Clinical Practice guidelines. All the study participants were >18 years old, provided signed informed consent. All enrolled volunteers underwent a blood drawing for research purposes. Moreover, data on age, sex, comorbidity, and employment status were collected by the study team. Starting from 15 April 2020, all the participants were screened to evaluate the presence of anti-SARS-CoV-2 antibodies. All subjects who were tested positive at anti-SARS-CoV-2 antibody evaluation or who developed a confirmed SARS-CoV-2 infection before 31 April 2020 were excluded from this study. Following this screening, we casually selected 1000 samples suitable for the targeted metabolomic analysis. The primary endpoint of this study was to assess the protective role of monolaurin against COVID-19 infection and the identification of the relevant cut-off. The 1000 volunteers whose samples underwent targeted metabolomic analysis were followed up for 6 months (1 May until 1 November 2020), and the development of SARS-CoV-2 infection (confirmed through molecular nasopharyngeal swabs in symptomatic individuals and direct contacts of infected ones, or via hospital monitoring, following the current healthcare regulation) was evaluated at two time points (3 and 6 months from enrollment).

### 4.2. Sample Preparation for Metabolomic Analysis

Circulating monolaurin was extracted from an aliquot of 30 µL of serum through 1 mL of ACN/IPA/water solution (3:3:2) (Merck, Darmstad, Germany). An internal standard (deuterated tridecanoic acid and glycine) was also added. After vortex and centrifugation steps (15 min at 14,500× *g*), samples were dried and then derivatized with 20 μL of methoxyamine hydrochloride in pyridine (20 mg/mL) at 80 °C for 20 min, followed by silylation with 90 μL of 99% N,O-Bis(trimethylsilyl) trifluoroacetamide (BSTFA) (Merck, Darmstad, Germany) at 80 °C for 20 min. The derivatization protocol was performed by adding 20 μL of methoxamine hydrochloride in pyridine (20 mg/mL) and N,O-Bis(trimethylsilyl) trifluoroacetamide (BSTFA) at 99%. After the spiking of the internal standard (hexadecane 0.1 ppm), samples were injected and analyzed with gas chromatography coupled with mass spectrometry. A calibration curve with the monolaurin standard was prepared for absolute quantification.

### 4.3. GC–MS Analysis

The GC–time-of-flight mass spectrometry (GC–TOF/MS) was performed using an Agilent 7890B GC system (Agilent Technologies, Santa Clara, CA, USA) and a Pegasus (BT) TOF–MS system (Leco Corporation, St. Joseph, MI, USA) with an Rxi–5 ms column (30 m × 0.25 mm × 0.25 μm, RESTEK, Centre County, PA, USA). Helium was used as the carrier gas at a flow rate of 1.00 mL/min. Samples were injected at 280 °C in splitless mode. Chromatographic conditions started at 40 °C, were held isothermally for 5 min, and then were ramped at 8 °C/min to 300 °C and held isothermally for 20 min. Mass spectrometry settings were the following: ion source temperature at 250 °C, electronic impact ionization (EI, 70 eV), scanning range 35/550 m/z, and extraction frequency of 30 kHz. Chromatograms were acquired in total ion current (TIC) mode. Absolute quantification of monolaurin was conducted using a calibration curve with concentrations ranging from 0.01 to 5 µg/mL.

### 4.4. Statistical Analysis

Data were analyzed using MedCalc^®^ Statistical Software version 20.014 (MedCalc Software Ltd., Ostend, Belgium). Continuous variables were presented as mean ± standard deviation. Comparisons between means were performed using a two-tailed Student’s *t*-test (univariate analysis). To determine the cut-off values for the survival curves, ROC curves were constructed based on monolaurin concentration levels and the presence or absence of infection at 3 and 6 months. Non-parametric methods were utilized for the ROC curves, and the cut-off was selected based on the Youden Index. Survival curves based on monolaurin concentration cut-offs were generated using the Kaplan–Meier model and compared using the log-rank test. For the Cox multivariate regression analyses, the assumptions of proportional hazards were verified using Schoenfeld residuals, and the overall model fit was evaluated using Harrell’s concordance index.

## Figures and Tables

**Table 1:** Comorbidities in the whole study cohort. Data are expressed as absolute numbers and percentages.

Comorbidity	Absolute Number	%
Arterial hypertension	90	9.0%
Thyroid diseases	73	7.3%
Previous neoplasm	54	5.4%
Cardiovascular diseases	28	2.8%
Diabetes mellitus	22	2.2%
Hypercholesterolemia	10	1.0%
Celiac disease	10	1.0%
Systemic connective tissue disorders	8	0.8%
Multiple sclerosis	8	0.8%
Chronic liver disease	5	0.5%
Rheumatoid/Psoriatic arthritis	4	0.4%
Ulcerative colitis	4	0.4%
Hereditary iron metabolism disorders	2	0.2%
Spondylitis	1	0.1%

**Table 2:** Sex, age, and clinical description of the whole study cohort. Data refer to the whole study population with respect to the uninfected and infected statuses at 3- and 6-month follow-ups.

	Total	Not Infected 3 Months	Infected 3 Months	Not Infected 6 Months	Infected 6 Months
	1000	979 (97.9%)	21 (2.1%)	974 (97.4%)	26 (2.6%)
Male	295 (29.5%)	286	9	286	9
Female	705 (70.5%)	693	12	688	17
Age (years) mean ± SD	46.4 ± 11.5	46.5 ± 11.4	42.2 ± 11.8	46.5 ± 11.5	43.2 ± 11.7
Rhinorrhea			5		8
Headache			2		2
Anosmia/ageusia			1		3
Pharyngodynia			1		1
Asthenia			2		3
Fever			3		5
Dyspnea			0		0
Gastroenteritis			0		0
Hospitalizations			0		0

**Table 3:** The table shows the univariate analysis of various comorbidities, sex, and age (categorized on the mean value), on infection risk at 3 and 6 months using Kaplan–Meier analysis, the chi-square, and the statistical significance value (*p*).

Variable	Chi-Squared (3 Months)	*p* (3 Months)	Chi-Squared (6 Months)	*p* (6 Months)
Age	0.748	0.38	0.005	0.94
Sex	0.159	0.68	0.348	0.55
Diabetes	0.687	0.40	0.358	0.54
Hepatic diseases	0.107	0.74	0.132	0.71
Epilepsy	0.021	0.88	0.026	0.87
Renal diseases	0.085	0.76	0.106	0.74
Dyslipidemia	0.215	0.64	0.267	0.60
Hypertension	0.006	0.93	0.055	0.81
Autoimmune diseases	0.913	0.33	0.592	0.44
Thyroid diseases	0.213	0.64	0.479	0.48
Cardiovascular diseases	0.614	0.43	0.762	0.38

**Table 4:** Cox proportional hazards analysis at 3 months. From the left, the values of the regression coefficient (b), the standard error (SE), and the significance (*p*) are reported. Harrell’s C-index 0.705, 95% confidence interval 0606 to 0.804.

Covariate	b	SE	*p*
Age	−0.037	0.020	0.066
Sex	0.118	0.471	0.801
Diabetes	−12.654	683.120	0.985
Hepatic diseases	1.158	1.144	0.311
Epilepsy	−12.153	974.355	0.990
Renal diseases	−13.055	2208.189	0.995
Dyslipidemia	−12.245	1061.020	0.990
Hypertension	−12.438	680.847	0.985
Autoimmune diseases	0.460	0.841	0.584
Thyroid diseases	1.225	1.068	0.251
Cardiovascular diseases	−0.390	1.028	0.704
Neoplasms	−12.423	402.289	0.975
Multiple sclerosis	0.797	0.780	0.306
Age	−12.411	815.401	0.987
Monolaurin 0.45 mcg/mL	0.969	0.444	**0.029**

**Table 5:** Cox proportional hazards analysis at 6 months. From the left, the values of the regression coefficient (b), the standard error (SE), and the significance (*p*) are reported. Harrell’s C-index 0.705, 95% confidence interval 0.606 to 0.804.

Covariate	b	SE	*p*
Age	−0.027	0.018	0.135
Sex	0.051	0.435	0.906
Diabetes	−12.440	626.480	0.984
Hepatic diseases	1.125	1.129	0.319
Epilepsy	−12.250	890.217	0.989
Renal diseases	−13.238	2020.154	0.994
Dyslipidemia	−12.362	970.706	0.989
Hypertension	−12.406	622.313	0.984
Autoimmune diseases	0.217	0.821	0.791
Thyroid diseases	1.01	1.058	0.335
Cardiovascular diseases	−0.5504	1.024	0.591
Neoplasms	−12.440	367.717	0.973
Multiple sclerosis	0.563	0.765	0.461
Age	−12.552	743.731	0.986
Monolaurin 0.45 mcg/mL	1.146	0.406	**0.004**

## Data Availability

Raw data are available upon request (corresponding author), for reasonable and ethical uses and requests, the data can be found on the Zenodo data repository (article title).
